# Ecological drivers of sexual size dimorphism in northern chamois

**DOI:** 10.1002/ece3.70310

**Published:** 2024-10-07

**Authors:** Rudolf Reiner, Luca Corlatti

**Affiliations:** ^1^ Berchtesgaden National Park Berchtesgaden Germany; ^2^ Chair of Wildlife Ecology and Management University of Freiburg Freiburg Germany; ^3^ ERSAF‐Stelvio National Park Bormio Italy

**Keywords:** body mass, fecundity, forest cover, sexual selection, ungulates

## Abstract

Male‐biased sexual size dimorphism (SSD) is common in ungulates. The dominant scenario for the evolution of ungulate SSD suggests that habitat openness leads to greater SSD by increasing group size and thus sexual selection through male–male competition for mates. At a more proximate level, adaptive changes in SSD may result from the plastic response of individuals to environmental variation. In this study, we used 161,948 body mass data from a seasonally size‐dimorphic species, the northern chamois *Rupicapra rupicapra*, to examine the role of forest cover and other environmental variables in the expression of SSD. Data were collected from individuals hunted in the Austrian Alps, grouped into 28 mountain ranges with different forest cover, geological substrate and population density. Population‐specific growth curves were fitted using monomolecular models, and SSD was calculated as the log‐transformed ratio of male to female asymptotic body mass. A path model in which environmental factors indirectly influenced SSD via male or female body mass suggested that SSD increased with increasing density via reduced female body mass and decreased on siliceous substrates via reduced male body mass. Forest cover was negatively associated with body mass in both sexes, but not with variation in SSD.

## Graphical abstract

Chamois are an interesting species to examine sexual size dimorphism (SSD) as they exhibit very little skeletal size dimorphism, and SSD in body mass is largely seasonal. We analysed 161,948 body mass data of northern chamois (*Rupicapra rupicapra*) in the Austrian Alps to explore environmental influences on SSD. Our findings show that SSD increased with higher population density through reduced female body mass and decreased on siliceous substrates through reduced male body mass, while forest cover negatively affected body mass in both sexes but did not alter SSD.

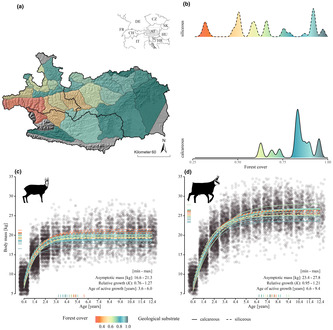

## 1 INTRODUCTION

Sex differences in body size are common in animals (Darwin, 1871). Although female‐biased sexual size dimorphism (SSD) is widespread, males are similar to or larger than females in most mammalian species (Andersson, 1994; Lindenfors et al., 2007; Ralls, 1976). From an evolutionary standpoint, SSD is the result of selective factors that act differently on the two sexes (Fairbairn, 1997): in terrestrial mammals, fecundity selection may influence female size (Ralls, 1976) because of the increasing costs of reproduction with large size (Lindenfors et al., 2007), while sexual selection tends to favour large males because of intra‐sexual competition for the monopolisation of females (Andersson, 1994; Darwin, 1871), although sexually selected traits may be countered by viability selection (Blanckenhorn, 2000; Delph, 2005). At a more proximate level, adaptive changes in SSD may result from the plastic response of individuals to environmental variation (Badyaev, 2002).

Among terrestrial mammals, artiodactyls show striking variation in SSD in body mass and horn/antler size (Bro‐Jørgensen, 2007); however, the latter may also depend on fighting style, and hereafter SSD will refer to differences in body mass. From the evolutionary standpoint, male‐biased SSD in ungulates is thought to be driven primarily by sexual selection acting on males (Andersson, 1994; Darwin, 1871; Loison et al., 1999). The dominant scenario for the evolution of SSD in this taxon is the eco‐evolutionary model of Jarman (1974), in which environmental and social factors interact to shape sexual dimorphism: increased habitat openness, leading to greater food dispersion, promotes larger group sizes. In turn, large groups increase male–male competition for access to females, favouring larger males and increased SSD. Jarman's hypothesis has been supported by several reviews of ungulate *taxa* (Corlatti & Lovari, 2023; Pérez‐Barbería et al., 2002; Szemán et al., 2021), with species‐specific variation due to the presence of counter‐selective forces, such as fighting style, which, depending on habitat characteristics, may favour variables other than body mass, such as speed or agility, and thus limit SSD (Blanckenhorn, 2000; Corlatti & Lovari, 2023). In addition to evolutionary processes, at a more proximate level SSD may be influenced by environmental factors that affect body mass variation in one or both sexes, possibly with different intensities for males and females (Badyaev, 2002; Post et al., 1999; Reiner et al., 2023; Sand et al., 1995), including habitat openness, climatic harshness, degree of seasonality, geological substrate, topography and population density (Andersson, 1994; Mason et al., 2011; Sæther, 1997; Sand et al., 1995; Vannini et al., 2021). These rapid environmentally induced changes may themselves become targets of selection.

**FIGURE 1:**
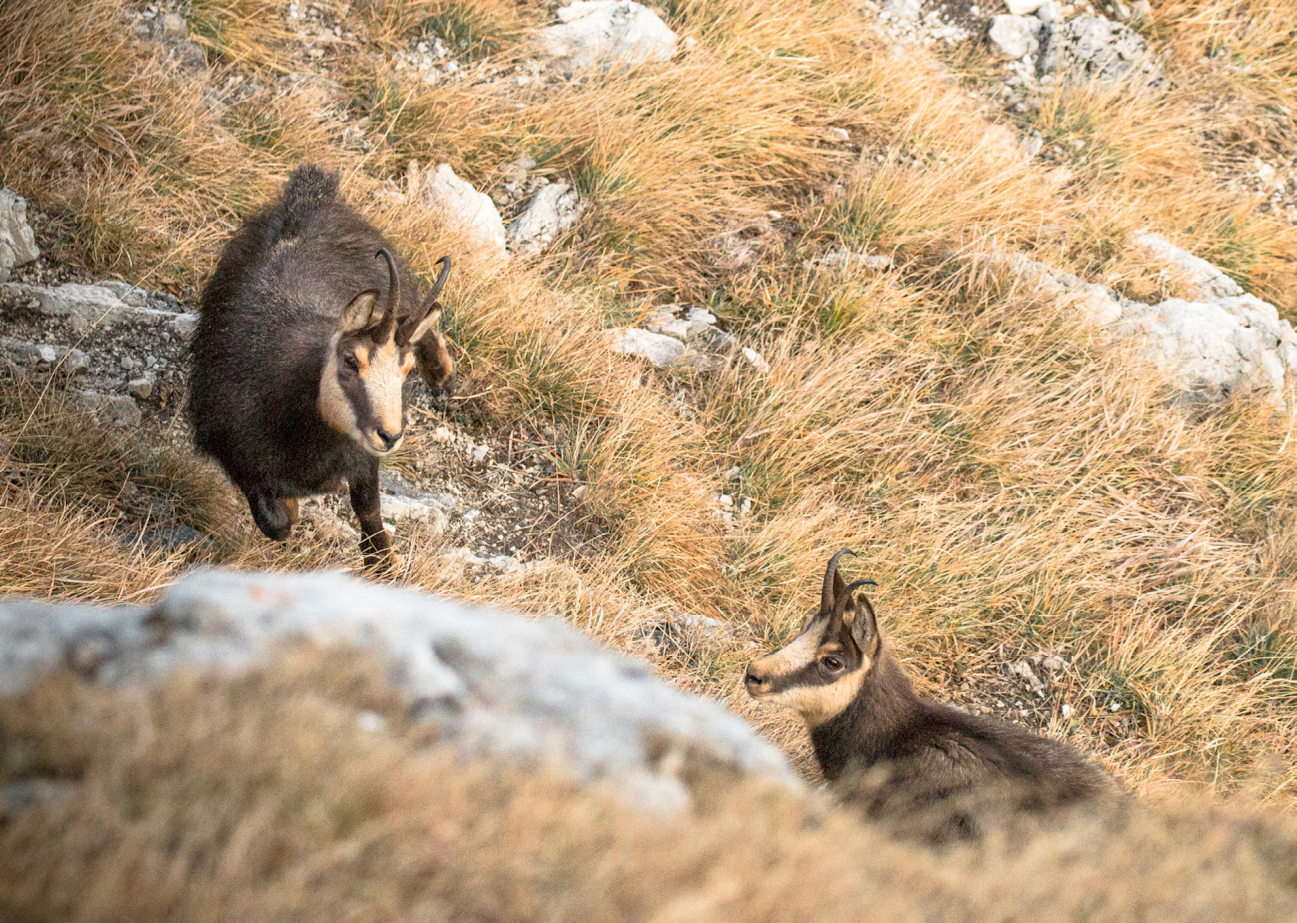
Male (left) and female (right) Alpine chamois. Chamois are an interesting species to examine sexual size dimorphism (SSD) as they exhibit very little skeletal size dimorphism, and SSD in body mass is largely seasonal.

The Northern chamois *Rupicapra rupicapra* is a medium‐sized goat antelope widely distributed in the mountainous regions of Europe and the Near East (Corlatti et al., 2022). The two sexes show little difference in overall appearance (Figure 1), but sexual dimorphism in body mass becomes apparent before the rutting season, with males weighing over 30% more than females (Bassano et al., 2003; Garel et al., 2009). As SSD is highly seasonal and dependent on mass accumulation during the summer (Rughetti & Festa‐Bianchet, 2011; Schröder, 1971), chamois is particularly suitable for investigating variation in sexual dimorphism as a function of environmental conditions. Rather than focusing on eco‐evolutionary dynamics, which are challenging to study within species, in this study we examine how populations experience environmental variation (Garel et al., 2006; Sand et al., 1995) to investigate the proximate ecological mechanisms or processes underpinning variation in SSD. Consistent with previous studies, we examine the direct effect of environmental variation on SSD (Garel et al., 2006; Post et al., 1999), but extend our investigation to the indirect pathways mediated by sex‐specific body mass to disentangle complex ecological pathways acting on individual components of SSD. We work under the null hypothesis of no association between environmental factors and SSD: as environmental conditions are known to affect chamois body mass (Reiner et al., 2023), the null hypothesis correspond to a situation where environmental factors either do not affect or affect both sexes equally. The null hypothesis may be rejected if environmental factors affect sex‐specific body mass differently: this recognises the potential for environmental pressures to drive divergent responses in male and female body mass, ultimately influencing patterns of SSD according to their respective reproductive roles and ecological niches. For example, in polygynous mammals, male growth is particularly affected by nutritional and environmental stress, thus limiting environmental conditions are expected to affect male body mass more than female body mass, ultimately decreasing SSD (Badyaev, 2002).

## 2 MATERIALS AND METHODS

### 2.1 Study area

**FIGURE 2:**
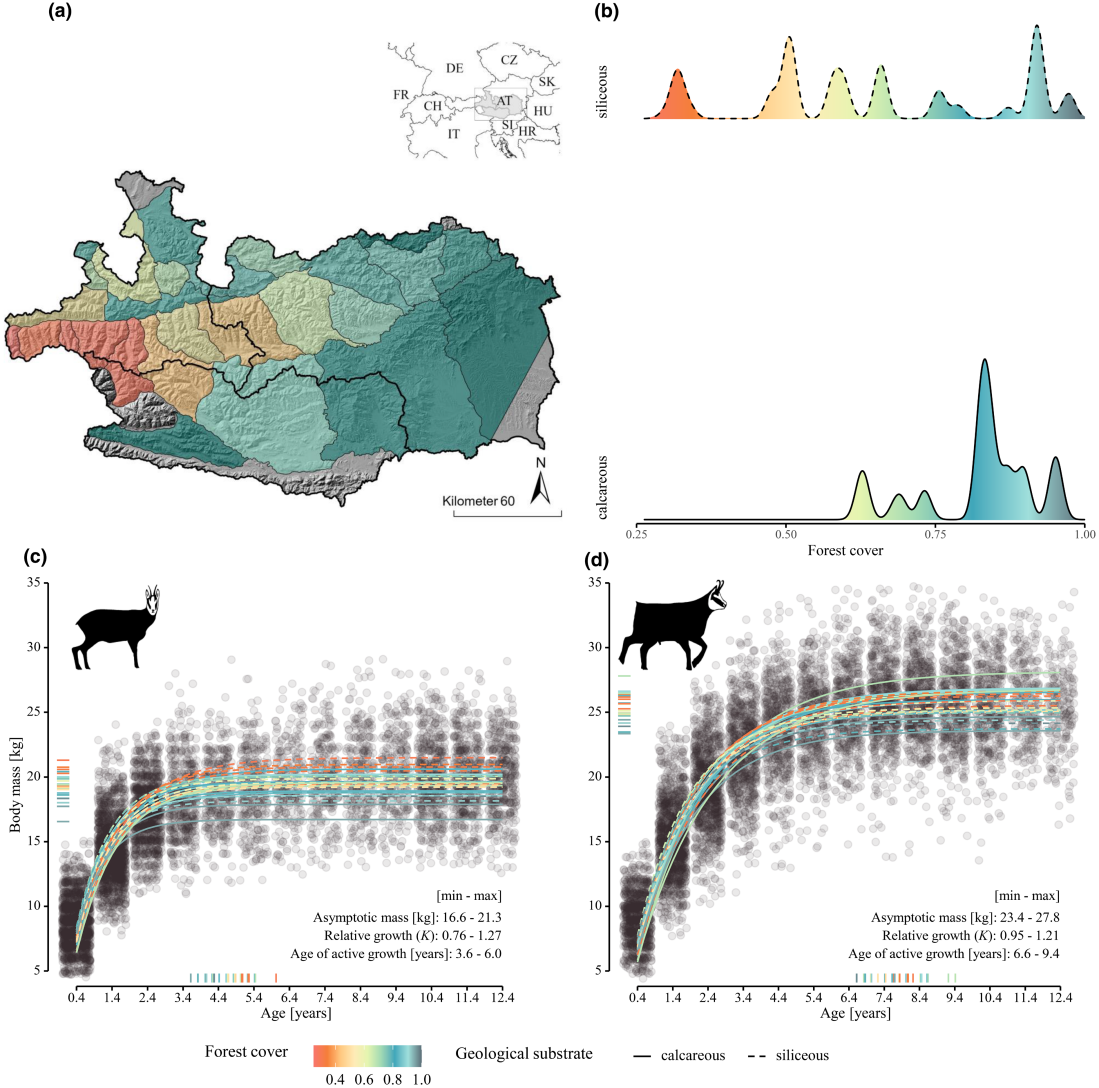
On top, (a) map of the 28 study populations in the Austrian Alps with (b) relative frequency of the 161,948 chamois harvested between 1993 and 2019 divided by forest cover and geological substrate. On bottom, monomolecular growth curves fitted to carcass mass data of (c) females and (d) males: Lines represent different populations; grey‐filled circles a subset (10%) of the data. Marks on the y‐axis indicate 99% of asymptotic body mass for each population, marks on the x‐axis the age at which animals reached 99% of asymptotic body mass. Minimum and maximum values of these indicators are shown within each panel. *K* indicates the relative growth rate.

Data were collected over an area of 13,600 km^2^ in the Austrian Alps (Figure 2a), in the provinces of Salzburg, Styria, and Carinthia, between 300 and 3500 m above sea level. The area includes chamois hunting grounds grouped into 28 chamois populations, which coincide with different mountain ranges (Grassler, 1984). The mountain ranges also differ in their geological substrate, with a calcareous type in the northern (*n* = 12) and southern limestone zones (*n* = 1), and a siliceous type in the central Alps (*n* = 15) (Grassler, 1984). Spruce *Picea abies* forests dominate in all mountain ranges. Beech *Fagus sylvatica*, Scots pine *Pinus sylvestris*, silver fir *Abies alba* and dwarf mountain pine *Pinus mugo* occur on calcareous soils, while silver fir and European larch *Larix decidua* occur on siliceous soils. Above the tree line (>1800 m a.s.l.), the habitat is dominated by alpine meadows.

### 2.2 Data collection

Chamois body mass data were obtained from individuals harvested by local hunters between 1993 and 2019 (*n* = 15 mountain ranges) and 1998 and 2019 (*n* = 13 mountain ranges). For each individual, eviscerated body mass (total mass minus all internal organs, blood and digesta, without head, with skin) was recorded to an accuracy of 0.5 kg. Age ranged between 0 and 12 years (animals older than 12 years were excluded because of low sample size in some populations) and it was estimated by horn ring counts (Schröder & von Elsner‐Schack, 1985). In total, we analysed body mass data from 161,948 individuals (77,172 females and 84,776 males). The average sex‐ratio of hunted individual across populations was 1.13 (SD = 0.18, based on data pooled over the entire period of study). As the hunting season runs from 16 July to 15 December in Salzburg and from 1 August to 31 December in Styria and Carinthia, and as chamois body mass is expected to change during the hunting season, to calculate SSD we first adjusted body mass values to Julian day 300, i.e. 27–28 October, which corresponded to the median shooting date, and to the pre‐rut period when body mass peaks. Body mass was adjusted by fitting additive models for each population, sex and age class (1/2/3/4–5/6–10/11–12 years: preliminary analyses showed that body mass patterns do not differ within these age classes) using the ‘mgcv’ package (Wood, 2017) for R 4.3.1 (R Core Team, 2023) in RStudio 2023.09.1 + 494 (Posit team, 2023), with body mass as the response and Julian date of shooting as the explanatory variable. Assuming that kids were born on 1 June (Garel et al., 2009), the adjusted body masses corresponded to individuals aged 0.4, 1.4, …, 12.4 years. For each population, we then modelled the age‐dependent growth of pre‐rut body mass using a monomolecular model: this approach, which assumes a monotonic increase with age and a decelerating growth rate from birth, appears to be appropriate for species with a high postnatal growth rate (Gaillard et al., 1997) and has previously been used to model body mass growth in chamois (Garel et al., 2009). The monomolecular growth curve equation was of the form:

$$E\left[\text{Body mass}|\mathrm{Age}\right]=\left({\text{Body mass}}_{\infty }-{\text{Body mass}}_{0}\right)\;e^{-K\left(\mathrm{Age}\right)}$$

where E[*Body mass*|*Age*] is the expected value of body mass at a given age (in years), *Body mass*_∞_ is the asymptotic mean body mass, *Body mass*_0_ is the mean body mass at birth, and *K* is the relative growth rate, which measures the exponential rate of approach to the asymptotic size (Ogle, 2016). Population‐specific monomolecular growth curves were fitted using a non‐linear least squares approach with the ‘nls’ function of the ‘stats’ package (R Core Team, 2023); for each population, initial values of *Body mass*_∞_ and *K* were obtained using the ‘FSA’ package (Ogle et al., 2023). As free estimation of all parameters in the monomolecular function resulted in predicted eviscerated body masses at birth above 4 kg, *Body mass*_0_ was fixed at biologically meaningful values (Caillet et al., 2006). In the absence of first‐hand data on body mass at birth, we used the closest available information, i.e. the population‐specific eviscerated body mass of males and females at 0.4 years of age. Specifically, to account for potentially different eviscerated body masses at birth between sexes and populations, we first linearly rescaled the mean eviscerated body mass values of 0.4‐year‐old individuals (separately for males and females across populations) so that they were assigned values between 1 and 2.4 kg (cf. Garel et al., 2009): 1 kg would correspond to the eviscerated body mass at birth (*Body mass*_0_) of the population with the lowest value of eviscerated body mass of 0.4‐year‐old individuals (calculated separately for males and females), and 2.4 kg would correspond to the eviscerated body mass at birth (*Body mass*_0_) of the population with the highest value of eviscerated body mass of 0.4‐year‐old individuals (calculated separately for males and females). Preliminary analyses suggested that the asymptotic body mass of both sexes was not much affected by whether *Body mass*_0_ was freely estimated or fixed, although fixed values allow for a better description of sex‐ and population‐specific growth curves. The resulting curves are shown in Figure 2c,d. The SSD was calculated for each population by log‐transforming the ratio of the asymptotic values of male and female body mass (Fairbairn, 2007).

Sexual size dimorphism variation was examined in relation to several ecological factors that are known to affect chamois body mass. To investigate the effect of habitat openness on chamois body mass (see Reiner et al., 2021) and SSD, we estimated forest cover as the relative area covered by forests (including deciduous, coniferous and mixed forests: Krofel et al., 2013) for each mountain range using Corine land cover data (Copernicus Land Monitoring Service, 2018) in ArcGIS Pro 2.6 (ESRI Inc, 2020). Mean forest cover ranged from 29.7% to 97.3% (Figure 2b), and did not change over the course of the study (Reiner et al., 2021). We also used information on geological substrate to explore potential effects on body mass, considering the influence of soil characteristics on vegetation communities, forage quality, and ultimately, growth patterns (see Mason et al., 2011): Chirichella et al. (2013) showed that soil type can influence horn growth in chamois, which suggests that it may also affect other traits, such as body mass. Finally, density dependence is known to affect several life history traits, including body mass (Bonenfant et al., 2009): we used the number of hunted chamois of all ages divided by the area of suitable habitat within each mountain range, averaged over the entire study period, as a proxy for population density (see details in Reiner et al., 2021). Although densities could vary over time within populations, a repeatability test conducted using the ‘rptR’ package (Stoffel et al., 2017) showed that differences between populations were consistent over the years (*R* = 0.649, *p*‐value < .001), thus we used a population‐specific density value.

### 2.3 Statistical analyses

We first tested for a direct relationship between SSD and forest cover, geological substrate, and log‐transformed density. Although sex‐specific asymptotic body mass values (and thus SSD) are based on estimates, sampling variance was low and moderately variable, thus we opted for an ordinary least squares approach over a weighted least squares approach (Lewis & Linzer, 2005); preliminary analysis also showed no major difference in the estimates between the two approaches. We tested for collinearity among explanatory variables using variance inflation factors (VIF) with the ‘vif’ function in the ‘car’ package (Fox & Weisberg, 2019); other potential environmental explanatory variables were excluded because they were highly collinear with forest cover (see 4. Discussion). We explored the individual contribution of explanatory variables to explained SSD variance using hierarchical partitioning with the ‘glmm.hp’ package (Jiangshan et al., 2022). To ensure comparability with the next analysis, parameter coefficients were estimated with *n* = 50,000 bootstrap samples with the ‘semEff’ package (Murphy, 2022), scaling all variables and adjusting for multicollinearity, so to obtain semipartial correlations, the unique relation between any explanatory variable and the dependent variable, with values ranging between −1 and +1; uncertainty was estimated using nonparametric bias‐corrected and accelerated confidence intervals (Murphy, 2022).

**FIGURE 3:**
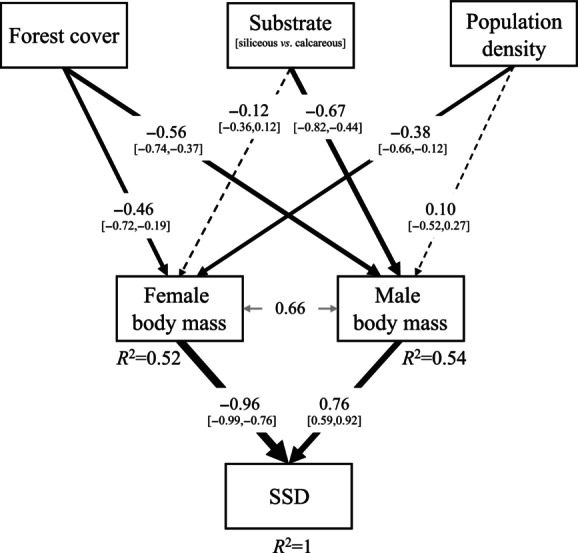
Pathways hypothesised to explain variation in sexual size (body mass) dimorphism (SSD) in northern chamois hunted in 28 populations of the Austrian Alps between 1993 and 2019. Female and male body mass are assumed to be mediators between environmental variables and SSD. Graphs show bootstrap estimates of semipartial correlation coefficients and 95% confidence bounds. Solid arrows indicate relationships with α < .05; dashed arrows relationships with α > .05. Line width indicate the effect size of the relationship. *R* ^2^ values refer to individual linear models.

To test for indirect effects of forest cover, geological substrate and density on SSD, we fitted a path model (Wright, 1934) using the R package ‘piecewiseSEM’ (Lefcheck, 2016). First, we constructed a directed acyclic graph reflecting biologically plausible causal chain hypotheses, in which forest cover, geological substrate and density directly influenced male and female body mass, and indirectly SSD (Figure 3). Given the definition of SSD, we assumed correlated errors between male and female body mass. We used the ‘lm’ function to fit each model in the causal network. Next, we assessed the overall goodness‐of‐fit of the hypothesised causal network using Fisher's *C* statistic, which combines the probabilities of all *k* independence claims implied by the model, and it is *χ* ^2^ distributed with 2*k* degrees of freedom (Shipley, 2016). Finally, we calculated direct, indirect and mediator effects as semipartial correlations using the ‘semEff’ package using *n* = 50,000 bootstrap samples as in the first model (Murphy, 2022). We inspected the residual distribution for each linear model using the ‘performance’ package (Lüdecke et al., 2021) (see Appendix 1).

## 3 RESULTS

**FIGURE 4:**
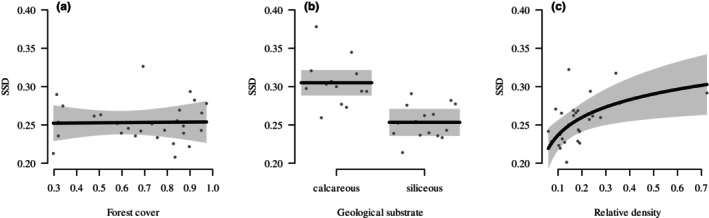
Marginal direct effects of (a) forest cover, (b) geological substrate and (c) relative density on sexual size (body mass) dimorphism (SSD) of northern chamois hunted in 28 populations in the Austrian Alps between 1993 and 2019. Dots indicate partial residuals. SSD was calculated as the log‐transformed ratio between male and female asymptotic body mass.

All linear models showed unsystematic residual distributions and no collinearity problems (VIFs < 2). The linear model testing the direct effects showed strong evidence for a lower SSD associated with siliceous substrate and higher SSD associated with density, while there was little to no evidence for a relationship between forest cover and SSD (Table 1 and Figure 4). The *R* ^2^ of the model was 62.8%: forest cover contributed 10.4% of the variance explained, while geological substrate and density respectively accounted for 55.8% and 33.8%.

**TABLE 1:** Model estimates of the direct relationships between northern chamois sexual size (body mass) dimorphism and forest cover, geological substrate and density in 28 populations of the Austrian Alps between 1993 and 2019.

Parameter	Coefficient	St. err.	95% confidence intervals	
			LCL	UCL
Intercept	0.000	0.000	0.000	0.000
Forest cover	0.010	0.108	−0.203	0.220
Substrate (siliceous vs. calcareous)	−0.477	0.103	−0.679	−0.278
Density	0.363	0.097	0.142	0.528

*Note*: The table shows bootstrap estimate of semipartial correlation coefficients with standard error (St. err.), and lower (LCL) and upper (UCL) 95% confidence levels.

The goodness‐of‐fit test for the hypothesised causal network supported the adequacy of the path model (Fisher's *C* = 10.059, d.f. = 6, *p*‐value = .122). Increasing forest cover had similar negative association with body mass in both sexes (Figure 3). Siliceous substrate was also negatively associated with body mass, but the effect was stronger in males than in females, while increasing density associated with reduced body mass for females, but not for males (Figure 3). SSD appeared to be more strongly associated with variation in female than male body mass (Figure 3). In line with the results of the linear model testing the direct effects of environmental variables, the path model showed that SSD was indirectly negatively associated with siliceous substrate and indirectly positively associated with increasing density, with no strong association with forest cover (Table 2). Overall, both sexes were important moderators of environmental drivers, i.e., the sum of all indirect pathways operating through body mass was similar between sexes (Table 2).

**TABLE 2:** Estimates of indirect and mediator effects for the causal network hypothesised to explain variation in sexual size (body mass) dimorphism in northern chamois in 28 populations in the Austrian Alps between 1993 and 2019.

Effect on SSD	Parameter	Coefficient	St. err.	95% Confidence interval	
				LCL	UCL
Indirect	Forest cover	0.020	0.089	−0.161	0.185
	Substrate (siliceous vs. calcareous)	−0.391	0.107	−0.623	−0.202
	Density	0.294	0.080	0.148	0.465
Mediator	Male body mass	−1.005	0.189	−1.393	−0.681
	Female body mass	0.927	0.267	0.528	1.372

*Note*: Indirect effects are the product of direct effects operating along causal pathways in each model, mediator effects are the sum of all indirect pathways operating through each individual mediator. The table shows, for each model, the type of effect operating on SSD, the parameter, bootstrap estimates of semipartial correlation coefficients, standard errors (St. Err.) and lower (LCL) and upper (UCL) 95% confidence levels.

## 4 DISCUSSION

The ecological causes of sexual dimorphism have long been the subject of research in animal ecology (Shine, 1989). In our study, population density and geological substrate were the most important direct predictors of SSD, while forest cover was loosely related to SSD. Very similar results were obtained when the same variables were assumed to be indirectly related to SSD, but path analysis provided a deeper understanding of the mechanisms underlying variation in SSD resulting from differential responses of male and female body mass to changing environmental conditions.

In our study, both male and female body mass were negatively associated with increased forest cover, resulting in no significant change in SSD. This, in turn supports the occurrence of environmental pressure acting similarly on both sexes, rather than changes in sexual selection on males only. Forested habitats covary with several environmental variables: for example, forests are typically found at lower elevations and have milder winter climates (less snow and higher temperatures) than open areas. As plant nutritional quality tends to increase in climatically harsher environments (Albon & Langvatn, 1992), forage quality, especially during the growing season, is lower in low‐elevation areas than in high altitude grasslands (Van Soest, 1994). In turn, forested habitat may represent a suboptimal habitat for chamois, in terms of forage quality, and decrease in body mass in forested areas is therefore not surprising: a similar result was also found in roe deer *Capreolus capreolus*, when more inviting alternatives to the forest were available (Hewison et al., 2009). Although our results do not support the prediction of Jarman's hypothesis, it is important to note that this study primarily focuses on ecological rather than evolutionary aspects, and that proximate environmental mechanisms operate on a shorter time scale than sexual selection (Hoekstra et al., 2001). Nevertheless, a few observations can be made: in the Japanese serow *Capricornis crispus*, the shift from forests to open areas is associated with increased group size (Takada & Minami, 2021). A similar pattern was observed in the Pyrenean chamois *Rupicapra pyrenaica* (Herrero et al., 2002). Large groups appear to favour the monopolisation of females, hence the transition from monogamy to polygyny in serow (Kishimoto, 2003; Takada et al., 2023). Whether this factor also leads to an increase in sexual size dimorphism, as predicted by Jarman's model (Jarman, 1974), remains unknown, although this would require sufficient genetic isolation between individuals inhabiting areas with different environmental features. Information on habitat‐specific variation in the mating system, particularly in terms of opportunity for sexual selection, is needed to understand whether variation in SSD would be expected over evolutionary time in chamois populations living under different environmental conditions.

Due to their higher energy requirements, males of polygynous species are generally expected to be more sensitive to resource limitation than females (Badyaev, 2002; Clutton‐Brock et al., 1982). Under food‐limited conditions, males may grow more slowly and reach smaller asymptotic body masses (Leberg & Smith, 1993). The fact that chamois are likely to be weakly polygynous (Corlatti et al., 2015) and are only seasonally dimorphic (Garel et al., 2009; Rughetti & Festa‐Bianchet, 2011; Schröder, 1971) suggests that male energetic requirements are lower than those of males in highly polygynous species. This may explain why the two sexes responded similarly to increased forest cover and why their body mass was limited by siliceous soils, although the effect was stronger in males than in females (Figure 3), thereby increasing SSD in areas with calcareous soils. The latter may occur because Alpine grasslands on calcareous soils are expected to be richer in species than grasslands on siliceous soils (Virtanen et al., 2003), possibly allowing males to grow larger (cf. Chirichella et al., 2013 on the effect of calcareous soils on chamois horn size).

Previous studies suggested that SSD decreases with increasing population density, probably because of resource limitation due to intraspecific competition, which affects males more than females in polygynous species (Leberg & Smith, 1993; Leblanc et al., 2001; Solberg & Sæther, 1994). On the contrary, in our study, chamois SSD was positively associated with increasing density, as only female body mass was strongly negatively affected. We do not have a clear explanation for this pattern, but some considerations can be suggested. In our study populations, the annual harvest rate is considered a good proxy for population density (Reiner et al., 2020, 2021, 2023), but it is a population‐specific value that cannot account for sex‐specific differences in distribution. The fact that male chamois generally use forests more than do females, hence they are more widespread (Nesti et al., 2010; Unterthiner et al., 2012), together with their lower level of sociality and the occurrence of territorial behaviour (Corlatti et al., 2015, 2022), might reduce the intraspecific competitive potential and possibly explain why a negative relationship was found between population density and body mass only in females. At the same time, we cannot rule out the possibility that higher densities may actually indicate more productive habitats capable of supporting large populations. If so, the observed density variation between populations would reflect habitat productivity rather than density variations within habitats of similar productivity. However, this would not explain why more productive sites would lead to females with lower body mass values. Although higher density values are expected to lead to greater body mass variations in males, the coefficient of variation of asymptotic body mass data was higher for females (5.2%) than males (4.2%), in line with the model results, and hierarchical partitioning analysis of the final linear model in Figure 3 suggests that most of the variance in SSD was explained by female body mass (67.7%) rather than by male body mass (32.3%). A thorough examination of SSD density dependence would require an analysis of SSD change over time. However, calculating annual growth curves for each population requires an extensive dataset: our approach stemmed from having only one SSD value per population, due to lack of sufficient data for several populations within each year, which precluded a dynamic assessment of population‐specific SSD variation over time. Another way to assess density dependence and the overall harshness of environmental conditions would be to look at site‐specific quality, measured, for example, by kid or yearling mass at a given date (Festa‐Bianchet et al., 2004). However, these metrics could not be used in our study due to collinearity with forest cover, which already serves as an indicator of habitat harshness.

Overall, while it remains unclear whether environmentally induced proximate changes in chamois SSD primarily affect the female or the male segment of the population, our study sheds light on this complex interaction. The exact proximate mechanisms leading to SSD variation are still unclear, highlighting the need for further replication studies to explore the role of environmental factors, particularly density dependence, in weakly dimorphic species. Importantly, our data, based on hunted individuals, provide valuable insights although potential occurrence of artificial selection (cf. Mysterud, 2011) and/or measurement errors cannot be excluded. Future studies focusing on live individuals could enhance our understanding by examining SSD in conditions unaffected by hunting practices (Fattorini et al., 2023), to strengthen our understanding of SSD dynamics in ungulates.

## AUTHOR CONTRIBUTIONS

**Rudolf Reiner:** Conceptualization (equal); data curation (equal); formal analysis (equal); investigation (equal); methodology (equal); supervision (equal); validation (equal); visualization (equal); writing – original draft (equal); writing – review and editing (equal). **Luca Corlatti:** Conceptualization (equal); data curation (equal); formal analysis (equal); investigation (equal); methodology (equal); supervision (equal); validation (equal); visualization (equal); writing – original draft (equal); writing – review and editing (equal).

## CONFLICT OF INTEREST STATEMENT

The authors have no conflict of interest to declare.

## Supporting information


Data S1:

## Data Availability

Dataset and R‐Script used for this analysis are available as supplementary material. Metadata are included in the R‐Script.

## APPENDIX 1

### 1.1

We present the residual diagnostic plots of the linear models fitted to investigate the direct and indirect effects of ecological variables on sexual size dimorphism (SSD) in Alpine chamois. The plots were generated using the ‘check_model’ function of the R package ‘performance’ (Lüdecke et al., 2021), which allows the testing of assumptions about:

1. The distributional family, through posterior predictive checks on real and simulated data (top left panel);
2. The linearity of the relationships (a straight line is assumed) (top right panel);
3. The homogeneity of variance (assuming equal dispersion of residuals) (middle left panel);
4. Influential observations, using Cook's distance (dashed lines) (middle right panel);
5. Multicollinearity between predictors (lower left panel);
6. The normality of residuals using Q–Q plot (points should fall along the line) (top right panel).

We first present the residual diagnostic plot for the linear model fitted to examine the direct relationship between SSD and forest cover, geological substrate and log‐transformed density (Figure S1). We then present residual diagnostic plots for the linear models fitted in the path model:

1. Path 1: linear model for the relationship between SSD and female and male body mass (Figure S2);
2. Path 2: linear model for the relationship between male body mass and forest cover, geological substrate and log‐transformed density (Figure S3);
3. Path 3: linear model for the relationship between female body mass and forest cover, geological substrate and log‐transformed density (Figure S4).
