# Supplementary material for: Ecological drivers of sexual size dimorphism in northern chamois
Source: Ecol Evol. 2024 Oct 7;14(10):e70310. doi: 10.1002/ece3.70310 (PMC11456770; doi:10.1002/ece3.70310)
Supplement: Supplementary file 1 — Data S1: [file ECE3-14-e70310-s001.zip › ece370310-sup-0002-FigureA2.pdf]

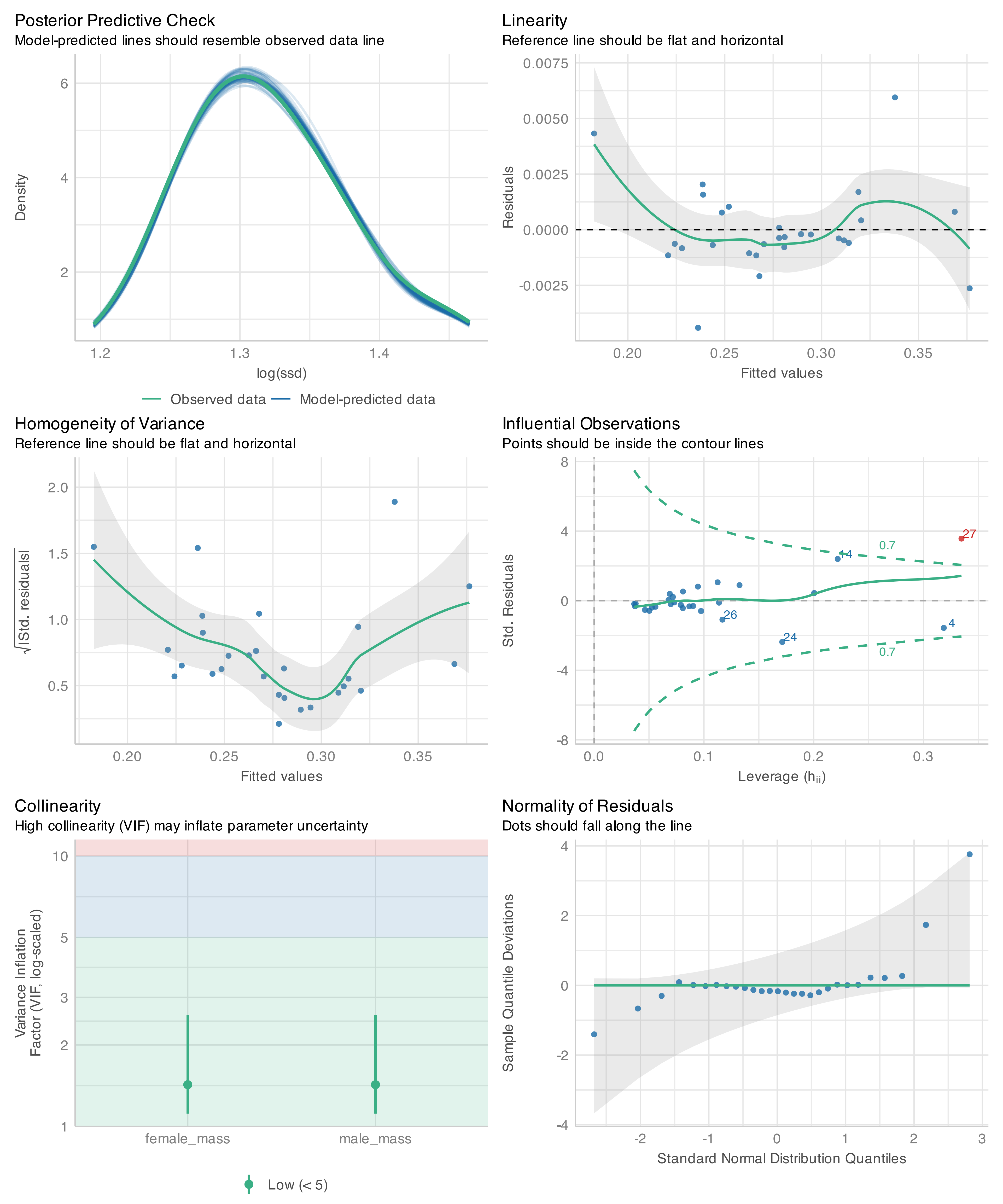

Posterior Predictive Check
Model-predicted lines should resemble observed data line
Density
log(ssd)
Observed data
Model-predicted data
Linearity
Reference line should be flat and horizontal
Residuals
Fitted values
Homogeneity of Variance
Reference line should be flat and horizontal
√|Std. residuals|
Fitted values
Influential Observations
Points should be inside the contour lines
Std. Residuals
Leverage (h_{ii})
0.7
27
14
26
24
4
Collinearity
High collinearity (VIF) may inflate parameter uncertainty
Variance Inflation Factor (VIF, log-scaled)
female_mass
male_mass
Low (< 5)
Normality of Residuals
Dots should fall along the line
Sample Quantile Deviations
Standard Normal Distribution Quantiles
