# Supplementary material for: Ecological drivers of sexual size dimorphism in northern chamois
Source: Ecol Evol. 2024 Oct 7;14(10):e70310. doi: 10.1002/ece3.70310 (PMC11456770; doi:10.1002/ece3.70310)
Supplement: Supplementary file 1 — Data S1: [file ECE3-14-e70310-s001.zip › ece370310-sup-0003-FigureA3.pdf]

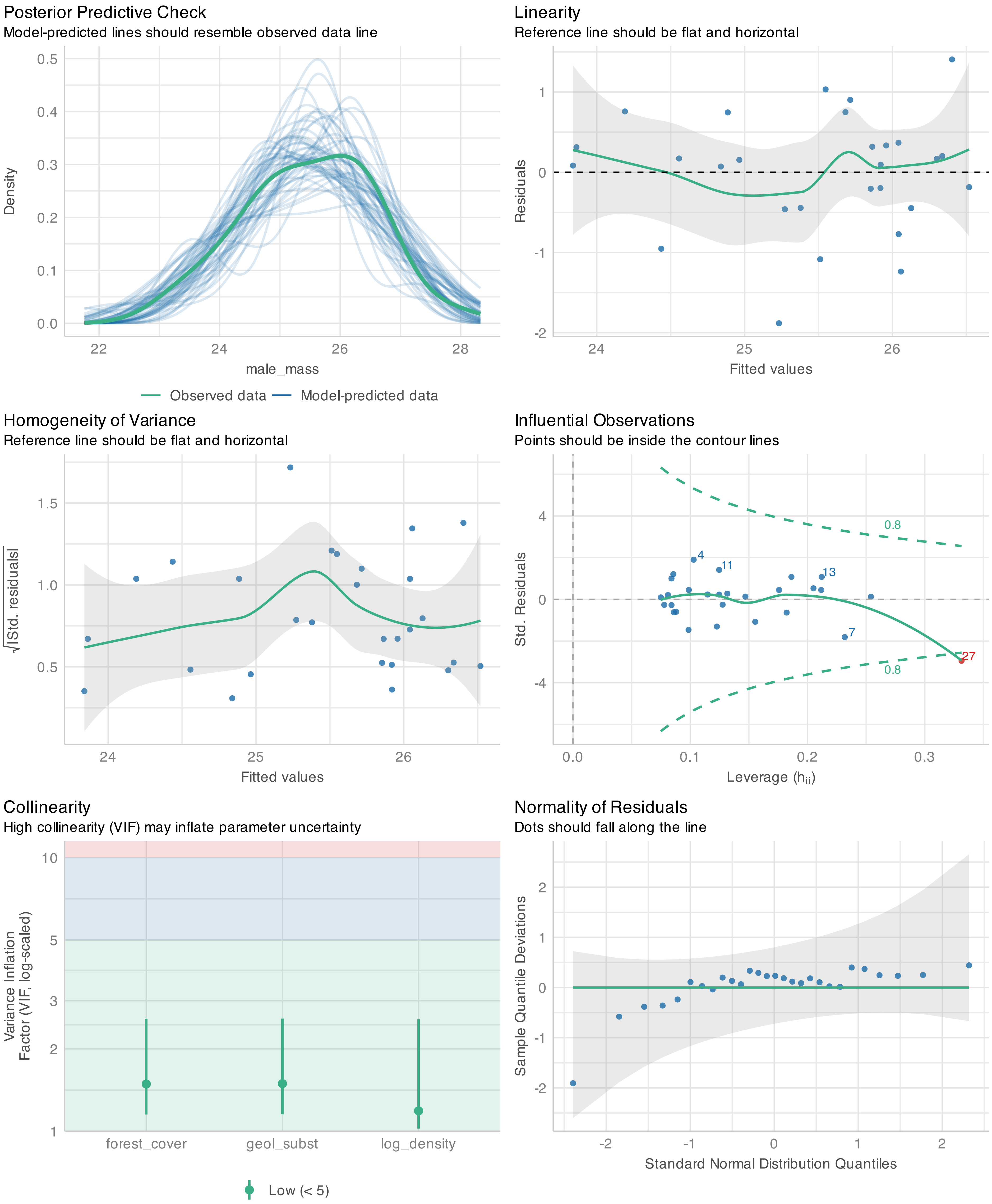

Posterior Predictive Check
Model-predicted lines should resemble observed data line
Density
0.0
0.1
0.2
0.3
0.4
0.5
22
24
26
28
male_mass
Observed data
Model-predicted data
Linearity
Reference line should be flat and horizontal
Residuals
-2
-1
0
1
24
25
26
Fitted values
Homogeneity of Variance
Reference line should be flat and horizontal
√|Std. residuals|
0.5
1.0
1.5
24
25
26
Fitted values
Influential Observations
Points should be inside the contour lines
Std. Residuals
-4
0
4
0.8
0.8
4
11
13
7
27
0.0
0.1
0.2
0.3
Leverage (hii)
Collinearity
High collinearity (VIF) may inflate parameter uncertainty
Variance Inflation Factor (VIF, log-scaled)
1
2
3
5
10
forest_cover
geol_subst
log_density
Low (< 5)
Normality of Residuals
Dots should fall along the line
Sample Quantile Deviations
-2
-1
0
1
2
-2
-1
0
1
2
Standard Normal Distribution Quantiles
